# Analytical and clinical characterization of an optimized dual monoclonal sandwich ELISA for the quantification of thymidine kinase 1 (TK1) protein in human blood samples

**DOI:** 10.1371/journal.pone.0275444

**Published:** 2022-10-06

**Authors:** K. K. Jagarlamudi, Swinkels L., Zupan M., Osredkar J., Venge P., Eriksson S.

**Affiliations:** 1 R&D Division, AroCell AB, Stockholm, Sweden; 2 Future Diagnostics, Wijchen, The Netherlands; 3 Blood Transfusion Center, Ljubljana, Slovenia; 4 University Medical Centre, Institute of Clinical Biochemistry, Ljubljana, Slovenia; 5 Department of Medical Sciences, Uppsala University, Uppsala, Sweden; 6 Department of Anatomy, Physiology and Biochemistry, Swedish University of Agricultural Sciences, Uppsala, Sweden; Lobachevsky University, RUSSIAN FEDERATION

## Abstract

Thymidine Kinase 1 (TK1) plays an important role in DNA precursor synthesis and serum TK1 activity has been used as a biomarker for prognosis and therapy monitoring of different malignancies. AroCell has developed a dual monoclonal antibody ELISA for determination of TK1 protein in clinical samples. The purpose of the study is to validate the ELISA analytically in relation to the gold standard, [^3^H]-deoxythymidine (dThd) phosphorylation assay for TK1 activity using sera from patients with different malignancies. The colorimetric TK 210 ELISA was validated analytically by assessment of precision, linearity, interfering substances, and stability. For the clinical validation, serum samples from patients with hematological malignancies (n = 100), breast cancer (n = 56), prostate cancer (n = 70) and blood donors (n = 159) were analyzed using TK 210 ELISA and TK1 activity by [^3^H]-deoxythymidine (dThd) phosphorylation assay. The sandwich TK 210 ELISA was highly specific for TK1 protein having a detection limit of 0.12 ng/mL, with a functional sensitivity of 0.25 ng/mL. Within-run CVs ranged from 5.5% to 10% and between-run CVs ranged from 5% to 15%. The ratio of observed to expected dilutional parallelism of 5 serum samples was in the range of 80–120%. Samples exhibited stability through four freeze/thaw cycles and 5 days at 4°C. Further, the ROC curve analysis showed that TK 210 ELISA and [^3^H]-dThd phosphorylation assay had similar sensitivity (62% vs 59%) in hematological malignancies. However, in the case of breast and prostate cancer sera, TK 210 ELISA had higher sensitivity (59% and 44%) compared to [^3^H]-dThd phosphorylation assay (47% and 25%) at a specificity of 98%. These data demonstrate that the dual monoclonal antibody based AroCell TK 210 ELISA is a robust, accurate and precise tool for measuring TK1 protein in different malignancies that can improve the clinical applications of TK1 as a biomarker in cancer management.

## Introduction

Thymidine Kinase 1 (TK1; ATP: thymidine 5´-phospho-transferase; EC 2.7.1.21) is a pyrimidine salvage pathway enzyme involved in DNA precursor synthesis. TK1 catalyzes the conversion of thymidine to deoxy thymidine monophosphate (dTMP), which is further phosphorylated to the corresponding triphosphates (dTTP) for incorporation into DNA [1]. In normal proliferating cells, TK1 is elevated during the late G1 phase, reaches a peak in the S phase, and then decreases during the M phase. However, in accelerated or uncontrolled proliferation, such as in cancer cells, TK1 remains elevated throughout the S and G2/M phases. This often leads to the release of large amounts of TK1 from the disruption of proliferating cells [2–5]. Furthermore, the close association with cell proliferation, serum TK1 (S-TK1) has been used as a biomarker for the prognosis, prediction, and monitoring of therapy as well as surveillance of malignant diseases. The use of commercially available TK1 activity assays has previously demonstrated that S-TK1 activity measurements can give information concerning prognosis and treatment monitoring mainly for patients with leukemia and lymphomas [6–11]. However, despite the substantial literature on TK1 as a proliferation biomarker, its routine use in clinical oncology has been limited. Contributing factors are that the TK1 activity assays have not been available on widely used diagnostic platforms and have shown low sensitivity with samples from patients with solid tumor diseases [12, 13].

Structure determinations of recombinat TK1 protein complexes have shown that active human TK1 are homotetramer of 25.5 kDa subunits. The crystalization experiments were performed with C-terminal truncated recombinant TK1 but the most likely structure of the 40 C-terminal aminoacid region has been modeled [14]. Biochemical experiments have demonstrated that this region is exposed and necessary for the degradation of TK1 during the M phase [4].

Several anti-human TK1 antibodies against peptides from the C-terminal region have been used to develop immunoassays as an alternative approach to TK1 activity assays [12, 15, 16]. Furthermore, TK1 protein determinations gives higher clinical sensitivity than the TK1 activity assays in sera from patients with solid tumors, most likely because of the presence of inactive forms of TK1 in the latter [12, 13, 17]. However, these inactive forms of serum TK1 still contains the C-terminal aminoacid sequence and so are measurable by immunoassays. Therefore, imunoassays measuring both active and inactive forms of serum TK1 are most likely superior tools for *in-vitro* diagnostics of different cancer diseases.

An ELISA for TK1 protein determination using two monoclonal antibodies with defined binding sites and complementarity-determining regions (CDRs) has been developed [18]. Here we describe the analytical validation of an optimized assay based on these anitibodies in the AroCell TK 210 ELISA which can be used for quantification of TK1 protein in human blood samples.

## Materials and methods

### Materials

Ninety–six well Microplates (Nunc Maxisorp-flat) were purchased from Thermo Fisher Scientific (Uppsala, Sweden). Wash buffer tablets (PBS-Tween 20 pH-7.4) were purchased from Medicago, (Uppsala, Sweden). Streptavidin-Horse Radish Peroxidase (Str-HRP), mouse IgG and bovine serum albumin (BSA) were purchased from Sigma Aldrich (Zwijndrecht, Netherlands), and the Tetramethyl benzidine (TMB) was from Neogen (Kentucky, USA).

### Preparation of human recombinant TK1

Recombinant human TK1 (Rec TK1) was used to prepare the calibrators. The full length human TK1 cDNA was cloned into the pET14b vector with an N-terminal His-tag as described previously [19]. The *E*.*coli* BL21 (DE3) cells were transformed using this recombinant plasmid. Then the transformed cells were grown in Luria-Bertani (LB) medium containing Ampicillin at 37°C to an optical density at 600 nm (OD_600_) of 1.2, then isopropylthio-β-D-galactopyranoside (IPTG) was added to a final concentration of 1mM. Cells were harvested by centrifugation and the cell pellets were lysed by sonication, the supernatant after centrifugation was kept for future purifications. The protein was purified by metal affinity chromatography on a Ni column as described [19]. The fractions were pooled and dialyzed followed by 0.22 μm filtration and then stored in a buffer containing 15 mM Tris-HCl, pH 7.6, 200 mM NaCl, 300 mM Imidazole, 2 mM MgCl_2_, 1 mM ATP, 5 mM DTT and 15% Glycerol at -80°C.

### Calibrator’s preparation

Purified Rec TK1 was diluted in a dilution buffer (15 mM Tris-HCl, pH 7.6, 200 mM NaCl, 300 mM Imidazole, 2 mM MgCl_2_, 1 mM ATP, 5 mM DTT and 15% Glycerol) and then added to a pre selected blood donor serum matrix to produce the calibrators concentrations in order to generate a dose response curve for the ELISA. In brief, the calibrator matrix was prepared by screening blood donor sera using TK 210 ELISA and selecting sera with low absorbance values (below 0.09 A_450 nm_). Then the selected donor sera were spiked with two different concentrations of recombinant TK1 (2.5 ng/mL and 10 ng/mL) for an initial pre-evaluation. The reference matrix with recombinant TK1 was used to determine the recovery, which should be between 70–130% compared to the reference matrix alone. Based on the recovery, the selected donor sera were used for further preparation of the final calibrator matrix. The recombinant TK1 was diluted in donor serum matrix to give a range from 0.5 to 18 ng/mL. There was no additional blank serum in our kit since we already subtracted its TK1 levels from the final calibrator concentration.

### Generation of monoclonal antibodies

The Mouse monoclonal IgG antibodies (Mabs) were purchased from Diatec Monoclonals (Oslo, Norway) and Genscript (Piscataway, NJ, USA). In breif, these antibodies were raised against the C-terminal region of TK1 by immunizing 6–8 weeks-old female Balb/c mice with selected TK1 peptides. Purified human TK1 C-31 peptides were coupled to BSA or KLH as previously described and used as immunogens [16, 18]. The immunization and maintenance of fusion murine cell lines from the best responding animals were performed according to GMP standards at Diatec Monclonals and Genscript. The supernatants were screened by ELISA procedures depending on high titers for the respective peptide conjugates and for human recombinant TK1 as well as for serum samples containing high TK1 levels. Based on these screening procedure for positive the hybridomas, two clones (i.e. Ar-1 and Ar-2 Mabs) were selected and subjected to larger scale production of pure antibody preparations.

### PepScan analysis

To determine the aminoacid regions to which Ar-1 and Ar-2 bound, a pepscan analysis was performed using synthetic peptides spanning aminoacids 193–226 from the C-terminal region of TK1 [16, 18]. A set of 14 octamerpeptides with six amino acids overlap and two aminoacid gaps were synthesized. These peptides were immobilized on streptavidin coated microplates (Thermo Fisher Scientific, Uppsala, Sweden) during 1h and then the plates were washed with 1% BSA in PBS-T. The antibodies were added to the wells at a concentration of 5μg/mL and incubated for 1h. The plates were washed again and incubated with an HRP conjugate. After a final wash, the absorbance values for each peptide were determined at 450 nm using TMB as substrate [18].

### Biosensor affinity analysis

The binding properties of Ar-1 and Ar-2 to recombinant TK1 were studied using a quartz crystal microbalance (QCM) biosensor (Attana A 200, Attana AB, Stockholm, Sweden). Recombinant TK1 (10, 5 and 2.5 μg/mL) was immobilized onto a LNB carboxyl chip by amine coupling using EDC and Sulfo-NHS [20]. Assays were performed with a flow rate of 10 μL/min during amine coupling and 25 μL/min during binding measurements at 22°C, using HBS-T (10 mM HEPES, 150 mM NaCl, 0.005% Tween 20, pH-7.4) as a running buffer. HBS-T injection was used as blank and sample injections were performed by the C-Fast auto sampler. The binding surface was regenerated after injection by applying 100 mM HCl for 60s and 20 mM NaOH for 30s. The data was analyzed via kinetic analysis using the Biacore T-200 evaluation software. The kinetic parameters including rate constants (K_on_, K_off_), dissociation constant (K_d_) and the maximum binding capacity (B_max_) were calculated.

### Validation studies

#### Analytical sensitivity

The AroCell sample dilution buffer (SDB) was used to determine the limit of the blank (LOB) of TK 210 ELISA. Sixty replicates of SDB measurements over 4 independent assays were used to calculate the LOB (95.5th percentile).To determine the limit of detection (LOD), 5 serum samples with TK1 protein levels in the range of 0.3 to 1 ng/mL were used. Sixty replicates were measured over 12 independent assays and the LOD was calculated: LOD = LOB + 1.653 * SD (Standard deviation of the serum sample measurements). The functional senstivity was defined as the concentration at which the mean interassay coefficient of varation (CV) is below 20%.

#### Precision

The precision of the TK 210 ELISA was evaluated by analyzing 5 calibrators (0.6, 1.28, 2.6, 6.3 and 16ng/mL) in duplicates in 44 independent assays. The intra-assay variation was assessed with 6 serum samples (concentration range of 0.3 to 3.1ng/mL) in quadruplicates from 5 independent assays. Inter-assay variation was assessed using 6 human serum samples (mean concentrations 0.53, 0.6, 0.78, 1.07, 2.46 and 3.14 ng/mL) tested in duplicates in 12 independent assays.

#### Linearity

Five serum samples from patients with hematological malignancies with high endogenous TK1 concentrations were diluted serially in a selected blood donor serum with a low TK1concentration and evaluated to assess the linearity of the assay. After dilution, the TK1 protein concentration in the sample was determined by subtracting the obtained TK1 concentration with the TK1 concentration from the blood donor sera that were used as diluent. The samples were serially diluted (1/2, 1/4, 1/8 and 1/16) and the accuracy of TK1 protein determination in each sample was compared with values determined at the 1/2 dilution.

#### Spiking and recovery

The recovery was evaluated by spiking recombinant human TK1 into blood donor sera with a low known TK1 concentration to increase the concentrations to 1.9, 3.5, 6.6, 9.8 and 16 ng/mL, respectively. The recovery of spiked TK1 protein at 5 different concentrations was measured in duplicates in 10 independent assays.

#### Plate homogeneity

Plate homogeneity was assessed by analysing all the positions of the 96 well ELISA plate with different calibrators having absorbance range from 0.3 to 1.0 AU (Arbitrary Units).

#### Hook effects

High TK1 protein concentrations were assayed to ensure lack of a “hook effect”. Thus, sera from healthy were spiked with 50 to 5000 ng/mL of recombinant TK1 protein and each concentration was assayed in quadruplicates.

#### Interference

The interfering effects of bilirubin, glyceryl triolate, hemolysis and albumin were investigated using four TK1 positive serum pools spiked with 20 mg/dL of bilirubin, 300 mg/dL of glyceryl triolate, 200–400 mg/dL of red blood cells and 60 mg/mL of albumin.

#### Sample stability studies

To evaluate the stability of serum TK1 protein as measured by TK 210 ELISA, clinical samples were stored at room temperature (RT, 22–25°C), 4°C, -20°C and -80°C. Twenty-one serum samples from patients with different malignancies were stored at RT and 4°C for 5 days and TK1 protein levels were measured on days 0, 1, 3 and 5.

A set of five serum samples from patients with hematological malignancies were stored at -20°C for a period of 8 weeks, serum TK1 protein levels in these sera were determined with the TK 210 ELISA on day 0 and after 1 week, 2 weeks, 4 weeks, and 8 weeks. Another set of four serum samples from patients with hematological malignancies were stored at -80°C for a period of 12 months and TK1 protein levels were analyzed in the sera at regular intervals day 0, and after 1, 2, 4, 8 and 12 months.

#### Freezing and thawing effects

Five serum samples from hematological malignancies were aliquoted and subjected to 4 cycles of freezing and thawing. The TK1 protein levels were measured after each freeze thaw cycle using the AroCell TK 210 ELISA.

### Ethics statement

Serum sample collection from patients with hematological malignancies was approved by the local ethical committee (2016/489), Uppsala University, Sweden.

Serum samples from breast and prostate cancer patients were purchased from Precision for Medicine (Massachusetts, USA) and Biotheme Research Solutions (Florida, USA). Samples were collected as de-identified diagnostic remainders excempt from Title 46, Title 21 and HIPAA IRB/ Consent requirements. Serum samples were collected under an IRB approved protocol or collected as consented donor samples from FDA licensed/registered facility following GMPs. The necessary procedures for obtaining the informed consent of donors were followed.

### Study population

A total of 159 serum samples from apparently healthy blood donors were obtained [N = 159; males (n = 117) and females (n = 42)] between 22 to 80 years of age, from the Blood Transfusion Centre, Ljubljana, Slovenia. Serum samples from 100 treatment-naïve patients with hematological malignancies i.e. Chronic Lymphocytic Leukemia (CLL, n = 46), Myeloid Dysplastic Syndrome (MDS, n = 15) Acute Myeloid Leukemia (AML, n = 11), Multiple Myelomas (MM, n = 10) and others [Chronic Myeloid Leukemia (CML, n = 5), B-cell Lymphoma (n = 4), Acute Lymphocytic Leukemia (ALL, n = 4), Marginal zone Lymphoma (n = 1), Mantle cell Lymphoma (n = 1), Acute Promyelocytic Leukemia (APL, n = 1), Small Lymphocytic Leukemia (SLL, n = 1) and Hairy Cell Leukemia (HCL, n = 1)] were obtained from the Department of Medical Sciences, Uppsala University Hospital, Sweden.

Another set of clinical samples from patients with confirmed breast cancer (n = 56) and prostate cancer (n = 70) were purchased from Precision for Medicine (Massachusetts, USA) and Biotheme Research Solutions (Florida, USA). All the serum samples were processed and stored at −20°C until they were analyzed with the AroCell TK 210 ELISA at the Dept of Anatomy, Physiology and Biochemistry, The Swedish University of Agricultural Sciences, Uppsala, Sweden.

### ELISA procedure

This section describes the ELISA assay procedure used in the validation process. The kit contains lyophilized calibrators covering the TK1 concentrations of 0.5–18 ng/mL and two lyophilized controls (High and Low) prepared in a human serum matrix. Calibrators, controls and serum samples were diluted 1:1 in sample dilution buffer (SDB), which was similar to the substrate buffers described previously [7, 21] in an uncoated microtiter (pre-treatment) plate, covered with a plate seal and incubated for 60 min at room temperature (RT).

The kit contains a 96 well microplate coated with an anti-TK1captured antibody (Ar-1), coupled at 4 μg/mL in 40 mmol/L carbonate-bicarbonate buffer pH 9.6 and which was blocked with 5% BSA in PBST. The antibody pre-coated plate was washed 4 times with wash buffer (phosphate buffer containing 0.05% Tween 20, pH-7.4, 350μL/well). After washing, diluted calibrators, controls and clinical samples were transferred in duplicates from the pre-treatment plate (100μL/well) to the antibody coated plate which was covered with a plate seal and incubated for 2h at RT with shaking at 650 rpm. After 4 washes, 100 μL of biotinylated anti-TK1 antibody (Ar-2, 3 μg/mL) was added to each well and the plate covered with a seal and incubated at RT for 1h. The wells were washed 4 times and then100 μL/well of streptavidin-HRP (130 ng/mL) was added, followed by plate seal and incubation for 30 min at RT. After a final wash cycle, 100 μL per well of TMB substrate was added and the plate incubated in the dark for 15 min. The reaction was terminated by adding 100 μL/well of stop solution (2N HCl) and the absorbance was measured at 450 nm with a microplate reader (Tecan Infinite M 200).

### Calculation of results

The TK1 protein concentrations in human serum samples were evaluated in duplicate. Each assay included a calibration curve with five concentrations, two controls (high and low) and SDB as a zero calibrator. The TK1 concentrations in serum samples and controls were determined by using absorbance (450 nm) of calibrators as standards in a 4-parameter logistic model (4-PL) by SoftMax Pro 7.1. The TK1 protein concentrations in the samples were expressed as ng/mL.

The acceptance levels of assay variation (CV) was set to < 20% for both intra and inter assay variations. Approximately 15% of the blood donor sera had TK1 levels below the limit of detection (LOD) and for statistical purposes, the TK1 protein concentrations in these samples were set to 0.12 ng/mL.

### Method comparison

The validated AroCell TK 210 ELISA was compared to the optimized [^3^H]-dThd phosphorylation TK1 activity assay. The optimized [^3^H]-dThd phosphorylation assay was performed as described previously [22].

### Statistical analysis

The TK1distributions in different groups were evaluated for normality using the D’Agostino and Pearson omnibus normality test. The TK1 protein and TK1 activity levels in both blood donors and patient sera followed non-Gaussian distributions. Consequently, the Mann-Whitney U test was used for comparing TK1 levels between groups, and unpaired *t*-tests were used to compare differences in TK1 levels between groups. For multiple groups comparison, The Kruskal-Wallis test followed by Dunn’s Multiple Comparison post-test was used, The Spearman correlation coefficient (*rs*) was used to determine correlations between the AroCell TK 210 ELISA and the [^3^H]-dThd phosphorylation assay. A receiver-operator characteristic (ROC) analysis was used to evaluate the performances of the TK 210 ELISA and [^3^H]-dThd phosphorylation assays using samples from patients with different malignancies. The statistical analyses were performed using Graph Pad Prism 5.0 (Graph Pad Software, La Jolla, CA, USA) and MedCalc 17.6 (Seoul, Republic of Korea). The level of significance was set at P<0.05.

## Results

### Characterization of the TK1 antibodies

**Fig 1:**
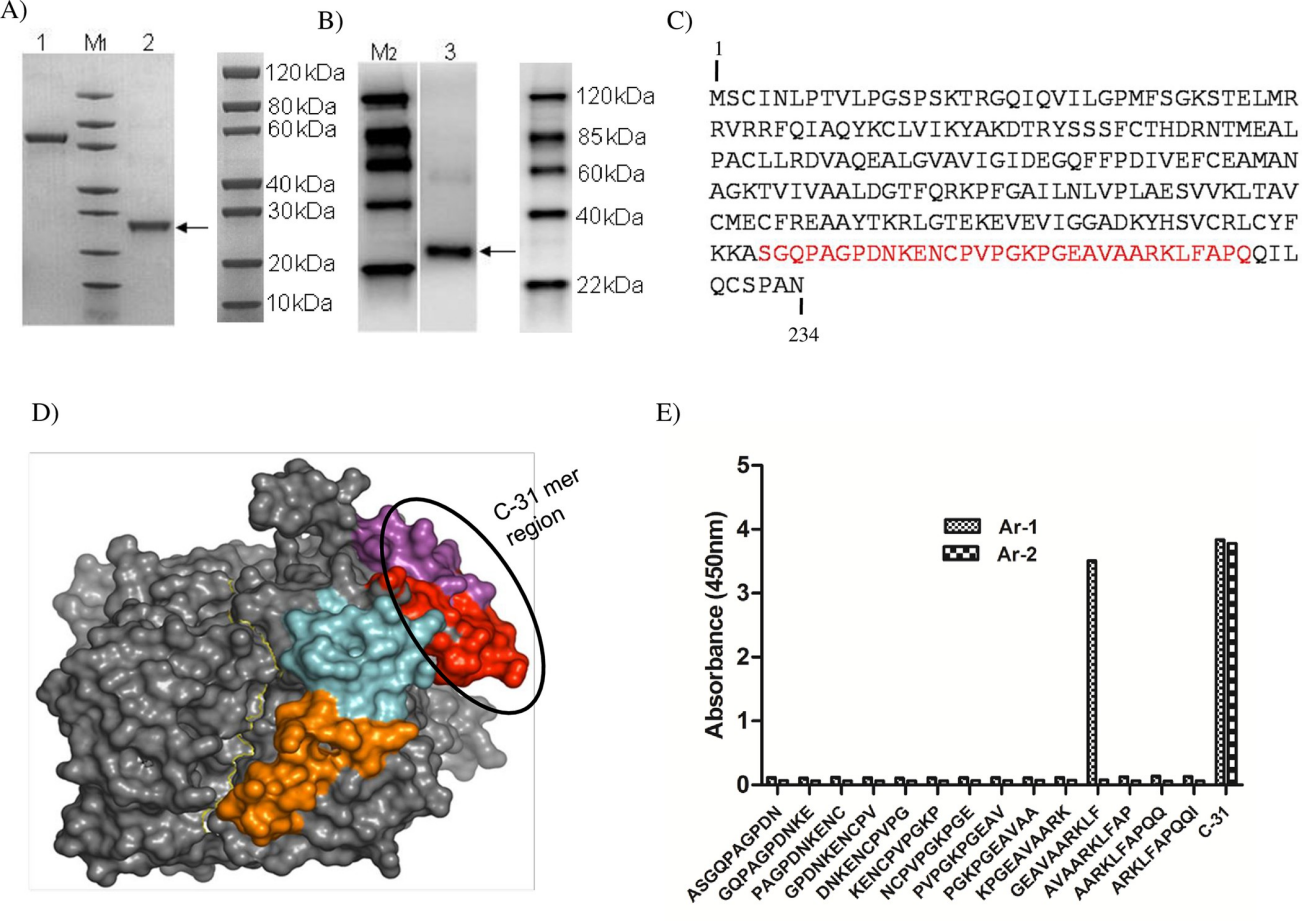
Characterization of TK1 antibodies. (A) An SDS-PAGE was performed with a 4% - 20% gradient gel for analysis of purified recombinant TK1, followed by Coomassie Blue staining, Lane 1: BSA (2.00 μg), Lane 2: Thymidine Kinase 1 (2.00 μg). (B) Western blot analysis of His-tag purified recombinant TK1 using Anti-His antibody (0.2 μg/mL), Lane 3: Thymidine Kinase 1 (C). The amino acid sequence of the human TK1 cDNA. The red color indicates the peptide sequences used for TK1 antibody production. (D) A model of the full-length human TK1 tetramer (produced by Martin Welin et al ref 14). The exposed KEN sequence and the C-terminal are shown in red and purple, and the lasso loop/active site is shown in light blue. (E) Pepscan analysis of the binding site of the anti-TK1 monoclonal antibodies with peptides from the 193–226 regions of TK1.

The purity of the recombinant TK1 preparation was determined by SDS-PAGE and was routinely > 95% (Fig 1A & 1B). The AroCell TK 210 ELISA is based on two monoclonal antibodies raised against the C-terminal region of human TK1 as shown in Fig 1C and the modelled 3D structure of this protein domain is shown in Fig 1D. The binding characteristics of Ar-1 and Ar-2 were determined using a Pepscan procedure with fourteen 10 amino acid peptides with two amino acid overlap, representing the region of amino acids 193–226. The results of the binding experiments are shown in Fig 1E and they demonstrated that Ar-1 bound to the GEAVVAARKLF peptide selectively, while Ar-2 only bound to the complete 31 mer peptide. This strongly indicates that Ar-1 bound to a linear epitope, while Ar-2 bound most likely to a conformational epitope, since it only reacted strongly with the entire 31-aminoacid peptide. These epitope mapping results are similar to those observed previously, where polyclonal antibodies reacting with this region (amino acids 210–225) of TK1 were analysed [16].

The binding kinetics of the two selected antibodies (Ar-1 and Ar-2) towards recombinant TK1 were characterized using an Attana QCM biosensor instrument. This analysis showed that the association velocity (K_on_) of Ar-1 was similar to that of Ar-2 but the dissociation velocity (K_off_) was faster for Ar-2 than that of Ar-1. Consequently, the calculated K_d_ was lower (0.7 nM) for Ar-1 compared to Ar-2 (4.2 nM) (Table 1). The results showed that the binding of Ar-1 and Ar-2 to recombinant TK1 were in the nM range and thus could be suitable for ELISA applications.

**Table 1:** Analysis data of antibodies and their kinetics using the Attana QCM-biosensor technology.

Antibody	Antigen	K_on_, Lmol^-1^.S^-1^	K_off_,S^-1^	K_d_, mol/L	Bmax (Hz)
Ar-1	Rec TK1	2.48×10^4^	1.6 ×10^-5^	0.65 ×10^-9^	107
Ar-2	Rec TK1	3.18×10^4^	1.3 ×10^-4^	4.12 ×10^-9^	78.3

### Validation results

**Fig 2:**
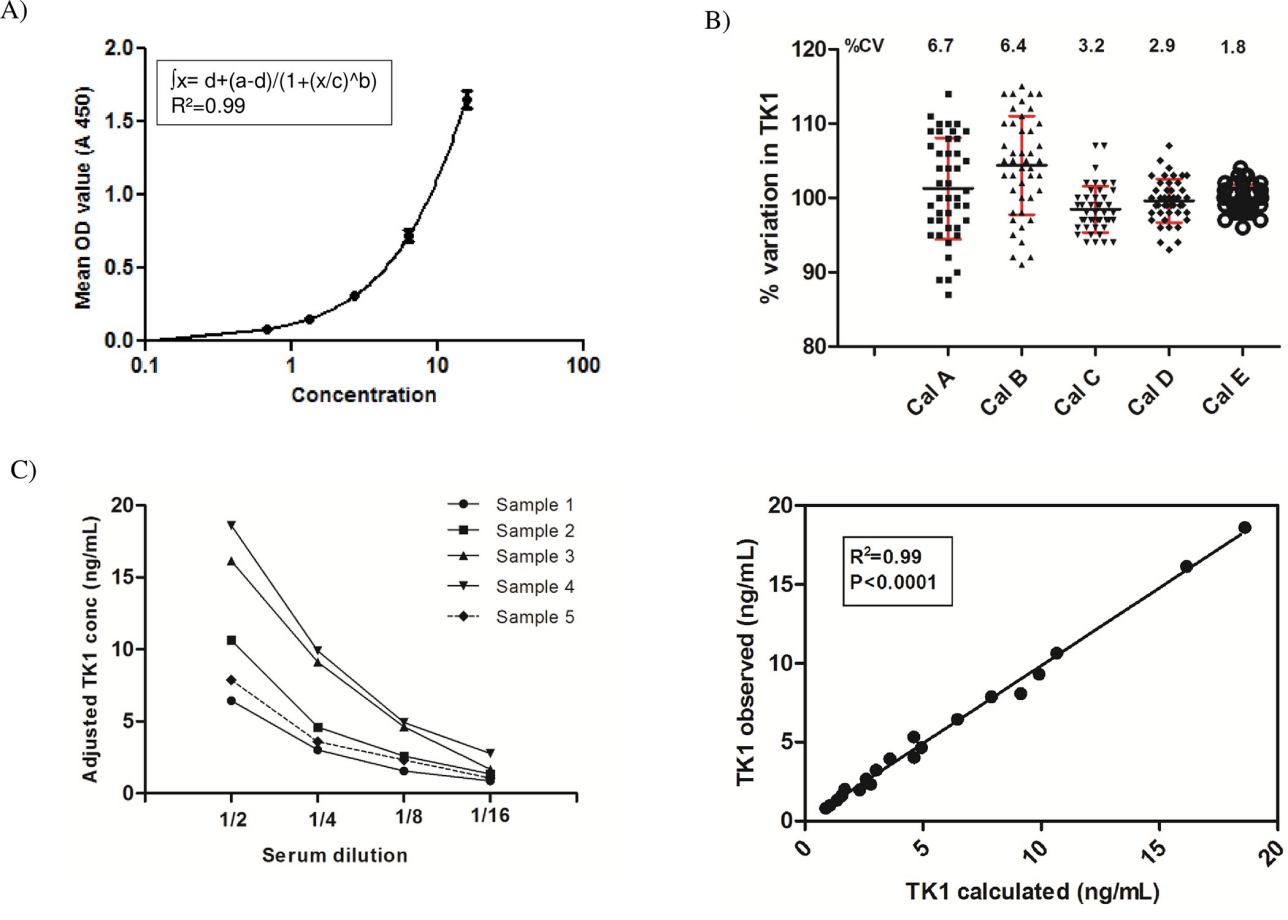
TK 210 ELISA standard curve and assay linearity. (A) A standard curve for TK 210 ELISA with calibrators (0–18 ng/mL) using 4-PL (non-linear regression program) in Graph pad prism. The limit of detection was 0.12 ng/mL. The error bars indicate the mean and standard deviation (SD) of 10 different experiments performed in duplicate. (B) The percentage change in calibrators from the precision assays. The error bars indicate the mean and standard deviation (SD) value of the calibrator. (C) The OD values of serially diluted serum samples measured by AroCell TK 210 ELISA. (D) The correlation between expected and observed TK1 protein levels in serially diluted samples.

The LOB of the assay was 0.08 ng/mL with an LOD of 0.12 ng/mL. The functional senstivity of the assay was detemined as 0.25 ng/mL and the upper limit of quantation was 18 ng/mL. The calibration curve comprised 5 concentrations of recombinant human TK1 in a serum matrix and using SDB as blank (0 concentration) as described in Materials and Methods. The concentration range was between 0 to 18 ng/mL recombinant TK1 as shown in Fig 2A. The back-calculated concentrations of the calibrators in the defined range met the acceptance criteria for recovery i.e. 80–120% and with an imprecision of better than 20%. The calibrators (0.6, 1.28, 2.6, 6.3 and 16ng/mL) were used in 44 independent assays to determine the imprecision. The between-run CV for the calibrators was in the range of 1.5 to 7.0%. The imprecision for each calibrator concentration is shown in the Fig 2B.

The intra-assay variation was assessed by analyzing six serum samples as quadruplicates in 5 independent assays. Nominal values of the samples were determined previously and were used as reference values (0.33 to 3.10 ng/mL) for intra-assay accuracy assessments. The intra-assay CV of the assay was within the range of 5.5 to 10% with a median of 6.9% based on 6 sera from cancer patients. Inter-assay variation was assessed by using another set of six serum samples (0.53 to 3.14 ng/mL) assayed in 12 independent assays (Table 2). The Inter-assay CVs was in the range of 5 to 15% with a median of 10.7%.

**Table 2:** Intra-and Inter assay variability of serum samples with AroCell TK 210 ELISA.

*Intra-assay variability*						
	**Sample 1**	**Sample 2**	**Sample 3**	**Sample 4**	**Sample 5**	**Sample 6**
Mean TK1 concentration (ng/mL)	0.60	0.33	0.49	0.69	1.68	3.04
N	5	5	5	5	5	5
SD	0.05	0.02	0.04	0.04	0.11	0.22
CV(%)	8.5	5.6	9.1	6.2	6.4	7.3
*Inter-assay variability*						
	**Sample 1**	**Sample 2**	**Sample 3**	**Sample 4**	**Sample 5**	**Sample 6**
Mean TK1 concentration (ng/mL)	1.07	3.14	0.60	0.78	0.53	2.50
N	12	12	12	12	12	12
SD	0.09	0.19	0.08	0.1	0.08	0.13
CV(%)	8.0	6.1	13.4	13.3	15	5.5

SD = standard deviation, CV = co-efficient of variation

The assay was linear on dilution down to 0.25 ng/mL and the measured values were within the range of 80–120% after dilution of a high endogenous TK1 sera with blood donor sera (Table 3). In case of clinical samples acceptable linearity was obtained at 4-, 8-, 16-fold dilutions (Fig 2C). A significant correlation was found between the expected and observed TK1 concentration after dilution (Fig 2D). The recovery of spiked TK1 was 87 to 105% for samples within the range of 1 to 16 ng/mL (Table 4).

**Table 3:** Dilution linearity of AroCell TK 210 ELISA.

			Meanadjustedconcentration	
	Dilution	OD values	(ng/mL)*	Accuracy (obs/exp×100)
Sample 1	1/2	0.624	6.45	
	1/4	0.364	3.02	93.2
	1/8	0.218	1.56	96.9
	1/16	0.179	0.88	108
Sample 2	1/2	0.969	10.65	
	1/4	0.508	4.61	86.6
	1/8	0.327	2.59	97.4
	1/16	0.219	1.36	102
Sample 3	1/2	1.346	16.14	
	1/4	0.893	9.13	113
	1/8	0.510	4.62	114
	1/16	0.247	1.68	119
Sample 4	1/2	1.491	18.6	
	1/4	0.952	9.91	106
	1/8	0.537	4.93	106
	1/16	0.344	2.78	119
Sample 5	1/2	0.747	7.88	
	1/4	0.417	3.59	91.1
	1/8	0.310	2.32	117
	1/16	0.189	1.06	107

*Mean adjusted concentration is TK1 protein levels in sera dilution after subtraction of diluent sera TK1 protein value.

**Table 4:** The % recovery of TK1 protein after spiking blood donor sera with human recombinant TK1.

	Expected concentration 1.90 ng/mL	Expected concentration 3.50 ng/mL	Expected concentration 6.60 ng/mL	Expected concentration 9.80 ng/mL	Expected concentration 16.0 ng/mL
**Mean TK1 concentration**	1.86	3.39	6.33	9.58	15.86
**Mean % Recovery**	98	95	94.6	98	99
**% Recovery (Range)**	88–101	91–100	90.1–102	90.4–105	95–103

The micro plate uniformity was tested at 2 concentrations that reflected low and high TK1 concentrations with A450 of 0.3 to 0.5 AU and at 0.5 to 1.0 AU. The CV was below 5% (2.5 and 2.1%) consistent with the abscence of significant row or column effects within the microplates. All samples with a concentration of TK1 exceeding 50 ng/mL were reported as overflow by the plate reader and no hook effect up to 5000 ng/mL was observed. The presence of high concentrations of hemoglobin (2 and 4 mg/mL) and albumin (60 mg/mL) lead to significant interference with the TK1 protein measurement but no interference was observed with bilirubin and triolates (S1 File).

### Stability of serum TK1

**Fig 3:**
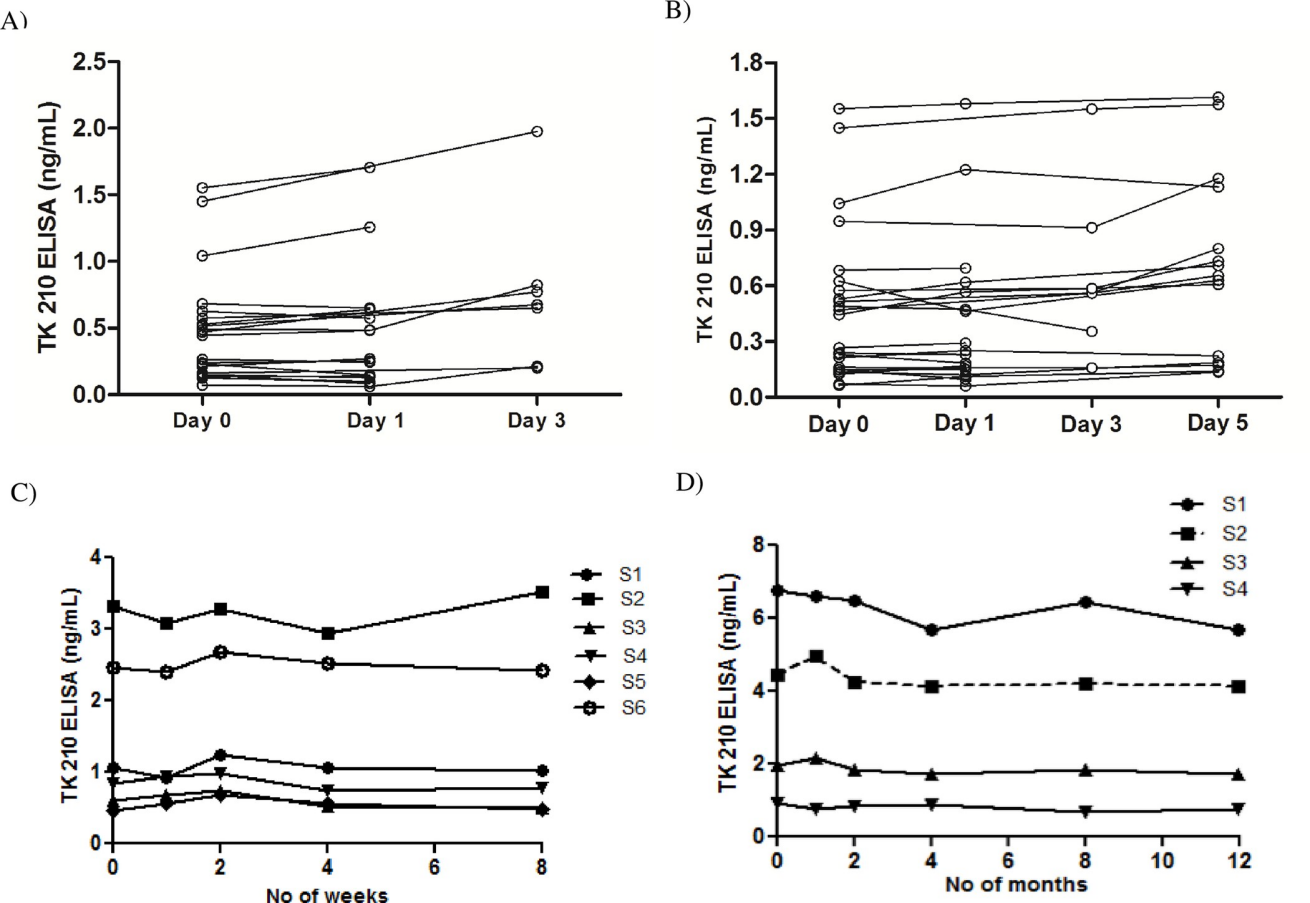
Stability of serum TK1 measured with AroCell TK 210 ELISA. (A) Serum TK1 protein stability at RT (22–25°C). (B) Serum TK1 protein stability at 4°C. (C) Serum TK1 protein stability of the clinical samples at -20°C. (D) Sample TK1 protein stability at -80°C.

A set of 21 serum samples were stored at RT and 4°C, the TK1 protein levels in the sera were determined on day 0, 1, 3 and 5. The results showed that TK1 protein in serum samples is stable at RT for 24 hours and there was no significant difference in median TK1 protein levels between day 0 and day 1. The TK1 protein levels showed significant variations at day 3 compared to day 0. Based on this data, TK1 protein in serum is stable at RT for 24 hours (Fig 3A). There was no significant difference in median TK1 protein levels in serum samples up to 5 days of storage at 4°C (Fig 3B). A few serum samples showed fluctuations after day 1 but it was not significant compared within-day variation on day 0 (within 20% CV). These results indicate that the TK1 protein in serum samples is stable at 4°C for at least 5 days.

A set of five serum samples from patients with hematological malignancies were stored at -20°C for a period of 8 weeks, serum TK1 protein levels in these sera were determined with AroCell TK 210 ELISA on day 0 and after 1 week, 2 weeks, 4 weeks, and 8 weeks. The % CV in TK1 protein at day 0 compared to 8 weeks was in the range of 4 to 12% (Fig 3C). These results demonstrate that serum TK1 is apparently stable at -20°C for at least 8 weeks.

Another set of four serum samples from patients with hematological malignancies were stored at -80°C for a period of 12 months and TK1 protein levels were analyzed in the sera at regular intervals day 0, and after 1, 2, 4, 8 and 12 months. The %CV in TK1 protein concentration ranged from 7 to 11% compared to day 0 values (Fig 3D). These results showed that serum TK1 protein is apparently stable for at least 1 year at -80°C.

#### Freezing and thawing effect

**Fig 4:**
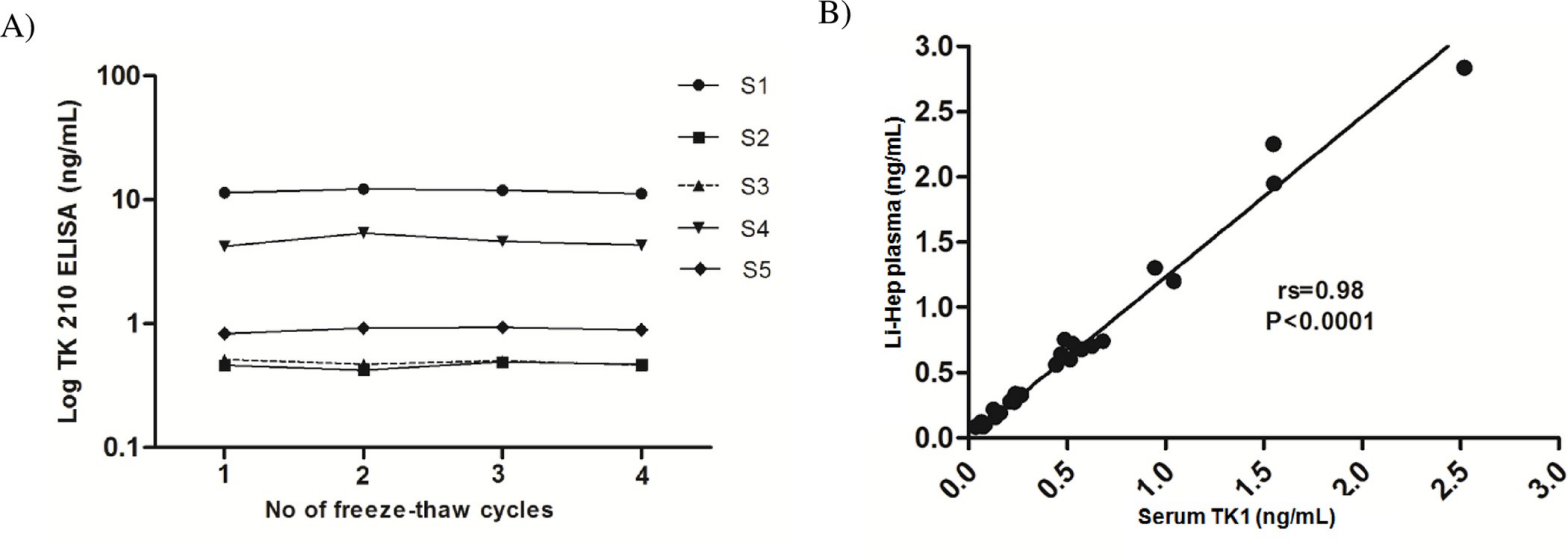
Effect of freeze-thaw cycles and sample type on TK 210 ELISA. (A) Effect of freeze and thaw cycles on the serum TK1 protein concentrations determined with AroCell TK 210 ELISA. (B) Correlation between serum TK1 and lithium heparin plasma TK1 protein levels in clinical samples.

Five serum samples from hematological malignancies were subjected to 4 cycles of freezing and thawing. The TK1 protein levels were measured after each freeze thaw cycle. Serum TK1 protein concentrations were minimally affected by up to 4 freeze-thaw cycles since the variation between runs was less than 15% (Fig 4A).

#### Comparison of serum and plasma TK1

TK1 protein levels in matching lithium (Li) heparin plasma samples and serum samples were assayed. There was a significant correlation between serum and Li-Heparin plasma samples (n = 21; *rs* = 0.98, P<0.0001) as shown in Fig 4B. The TK1 protein levels in Li-Heparin plasma were 10–15% higher compared to serum TK1 protein levels in the same samples based on comparisons of more than 100 sera and Li-Heparin samples from the same healthy and patient samples, respectively. This demonstrates that the AroCell TK 210 ELISA can be used to measure TK1 protein determinations in both serum samples as well as lithium-heparin plasma samples.

### Clinical evaluation

The AroCell TK 210 ELISA was used to determine the TK1 protein levels in serum samples from blood donors and patients with different malignancies.

#### Control group

**Fig 5:**
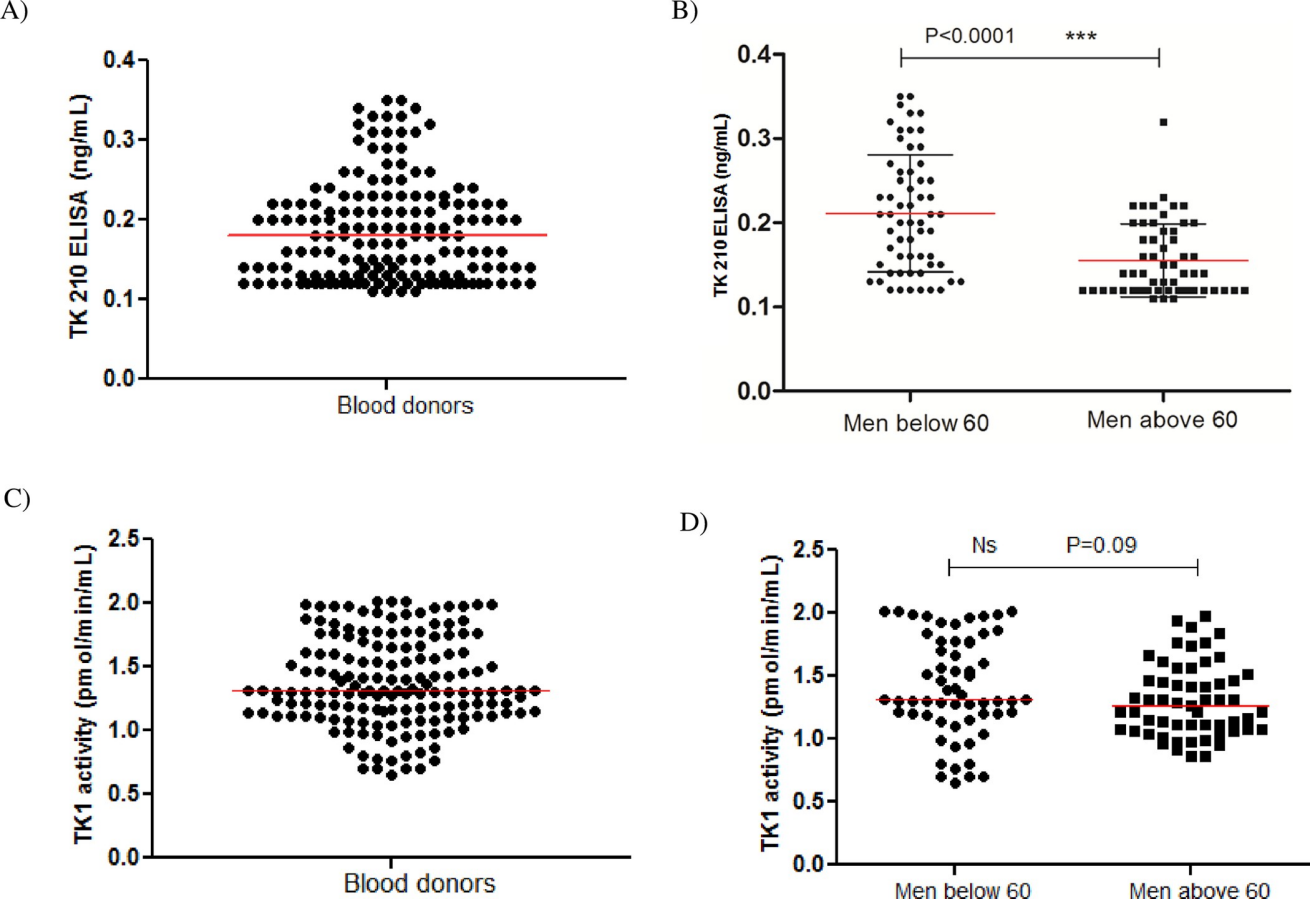
The distribution of serum TK1 protein and TK1 activity in blood donor samples (n = 159). (A) Serum TK1 protein distribution in blood donor sera (n = 159) (B) A comparison of TK1 concentrations in sera from blood donor men below 60 years (●) and men above 60 years (■). The error bars indicate the median (red). (C) Serum TK1 activity levels in blood donor samples (n = 159). (D) Comparison of TK1 activity in sera from blood donor men below 60 years (●) and men above 60 years (■). The error bars indicate the median (red).

Both TK1 protein and TK1 activity levels in 159 serum samples from blood donors (117 men and 42 women) were analyzed. The concentrations of TK1 ranged from 0.12 to 0.35 ng/mL (Fig 5A). The cut-off value estimated as the upper 98 percentile was 0.35 ng/mL, and the median value was 0.18 ng/mL. There was no significant difference in TK1 protein levels between men and women (P = 0.21). However, the TK1 protein levels were significantly higher in men aged below 60 years (n = 60) (Mean ± SD = 0.21±0.07 ng/mL; Median = 0.21) compared to older men (n = 57) (Mean ± SD = 0.16±0.04 ng/mL; Median = 0.14) (P<0.0001, Fig 5B). The STK1 activity levels in blood donor sera were in the range of 0.65–2.02 pmol/min/mL (Fig 5C) (mean ± SD = 1.38±0.36 and median = 1.30). In contrast to TK1 protein, there was no significant difference in the TK1 activity values in men below 60 years (n = 60: Mean ± SD = 1.44±0.39 pmol/min/mL; Median = 1.40) compared to older men (n = 57: Mean ± SD = 1.30±0.29 pmol/min/mL; Median = 1.27) (Fig 5D).

#### Patients with hematological malignancies

**Fig 6:**
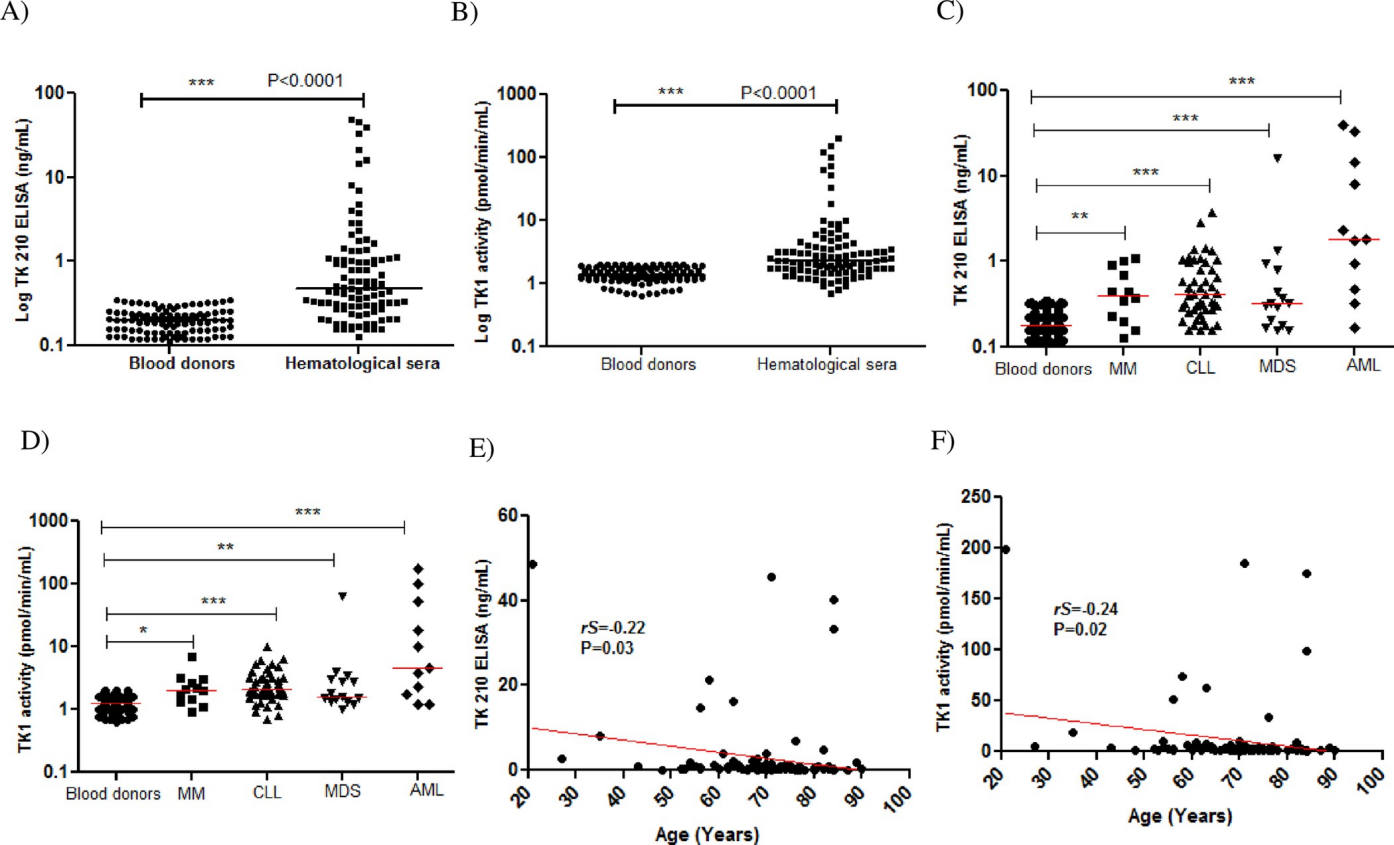
TK1 protein and TK1 activity levels in sera from patients with hematological malignancies. (A) TK1 protein distributions in sera from blood donors (●) and patients with hematological malignancies (■). (B) TK1 activity levels in sera from blood donors (●) and patients with hematological malignancies (■). (C) Comparison of TK1 protein levels in sera from blood donors (●), MM (■), CLL (▲), MDS (▼). and AML (▪). (D) Comparison of TK1 activities in sera from blood donors (●) MM (■), CLL (▲), MDS (▼). and AML (▪). The error bars denote median. (E) Correlation between TK 210 ELISA (ng/mL) and age (years) in HM patients. (F) Correlation between TK1 activity and age (years) in HM patients.

The TK1 protein levels in sera from patients with different hematological malignancies (n = 100: range 0.2 to 48.5 ng/mL, mean±SD = 3.05±8.65 ng/mL; Median = 0.48) were significantly higher compared with blood donors (P<0.0001, Fig 6A). The TK1 activity values were in the range of 0.7 to 199 pmol/min/mL (mean±SD = 10.7±30.4 and median = 2.28), which was significantly higher than in sera from blood donors (P<0.0001, Fig 6B). Furthermore, the TK1 protein levels in sub-classified HMs, such as multiple myelomas (MM, n = 12; mean ± SD = 0.51±0.34 and median = 0.41 ng/mL), chronic lymphocytic leukemia (CLL, n = 48; mean ± SD = 0.65±0.68 and median = 0.41 ng/mL), myeloid dysplastic syndrome (MDS, n = 16; mean ± SD = 1.50±4.1 and median = 0.35 ng/mL) and acute myeloid leukemia (AML, n = 12; mean ± SD = 9.40±14.1 and median = 1.50 ng/mL) were significantly higher compared to blood donors (Fig 6C). Similarly, the TK1 activity levels in sub groups were also significantly higher in MM (n = 12; mean ± SD = 2.4±1.6 and median = 2.1 pmol/min/mL), CLL (n = 48; mean ± SD = 2.7±1.7 and median = 2.2 pmol/min/mL), MDS (n = 16; mean ± SD = 6.1±15.7 and median = 1.70 pmol/min/mL) and AML (n = 12; mean ± SD = 33.5±55.7 and median = 4.60 pmol/min/mL) compared to blood donors as shown Fig 6D. There was no significant difference in TK1 protein or TK1 activity between women and men in patients with HM (S2 File). However, a significant negative correlation was found between age and both TK1 protein (*rs* = -0.22, P = 0.03) (Fig 6E) and TK1 activity (*rs* = -0.24, P = 0.02) (Fig 6F).

#### Patients with breast cancer

**Fig 7:**
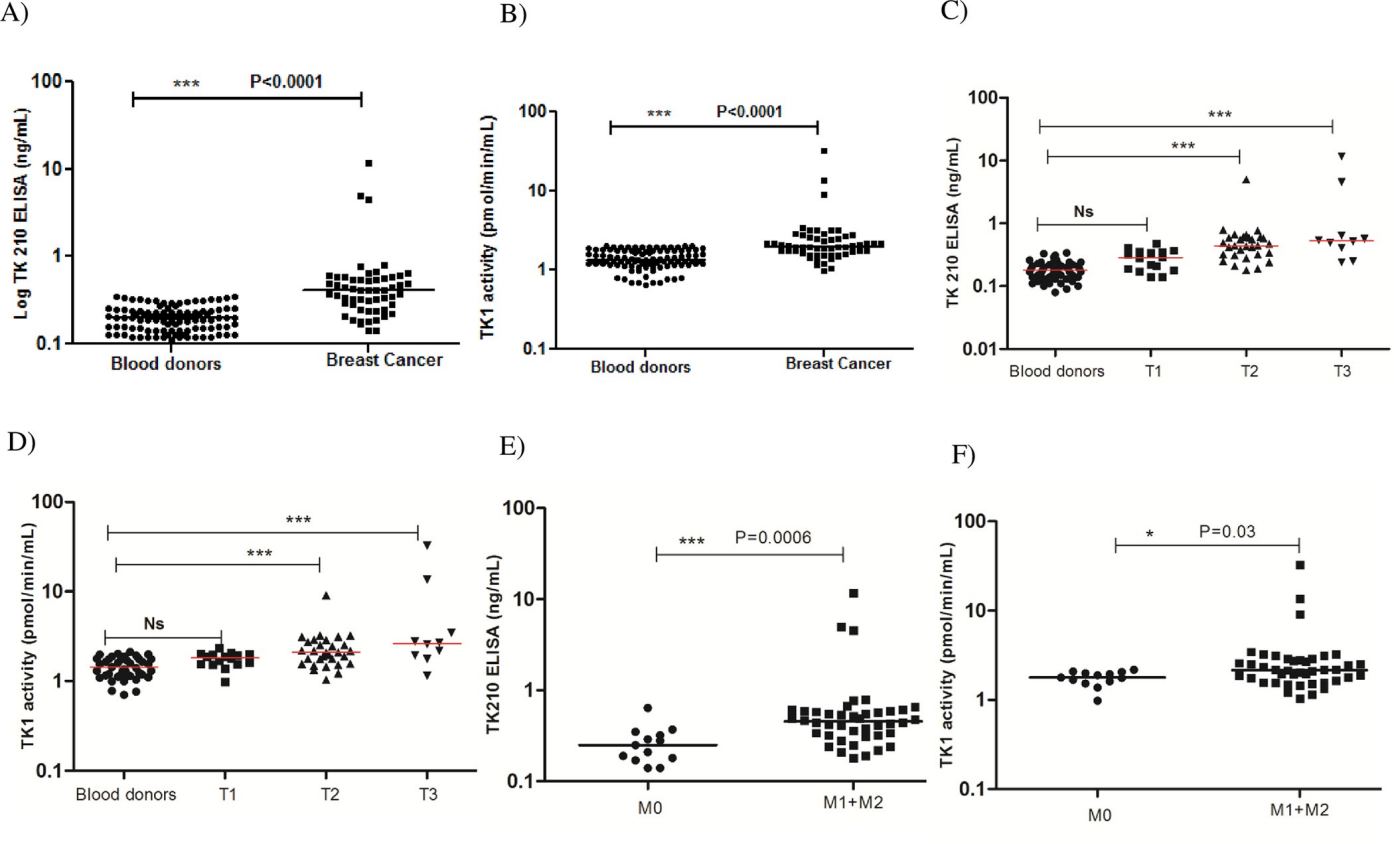
Distribution of TK1 protein and TK1 activity values in sera from patients with breast cancer. (A) TK1 protein values in sera from blood donors (●) and patients with breast cancer (■). (B) TK1 activity levels in sera from blood donors (●) and patients with breast cancer (■). (C) Comparison of TK1 protein levels in sera from blood donors (●), T1 (■), T2 (▲) and T3 (▼) breast cancer patients. (D) Comparison of TK1 protein levels in sera from blood donors (●), T1 (■), T2 (▲) and T3 (▼) breast cancer patients. (E) Comparison of TK1 protein levels in patients without metastasis (M0) (●) and patients with metastasis (M1+M2) (■). (F) Comparison of TK1 activity values in patients without metastasis (M0) (●) and patients with metastasis (M1+M2) (■). The error bars denote median.

In breast cancer sera (n = 56), the TK1 protein levels were in the range of 0.14–11.7 ng/mL (mean ± SD = 0.75±1.70 and median = 0.41) and were significantly higher than levels in sera from blood donors (Fig 7A). Similarly, to TK1 protein, the TK1 activity values in breast cancer patients were significantly higher levels compared with blood donors, and they are ranging from 0.98 to 32.5 pmol/min/mL (mean ± SD = 2.92±4.44 and median = 1.98; Fig 7B). In breast cancer patients, there was no significant correlation between TK1 protein (or) TK1 activity in relation to age (S3 File). The breast cancer sera were further classified into 3 groups based on tumor size (T), i.e., T1, T2 and T3, the TK1 protein levels were significantly higher in women with T2 (n = 31; mean ± SD = 0.63±0.84 and median = 0.45 ng/mL), and T3 (n = 11; mean ± SD = 2.00±3.60 and median = 0.53 ng/mL) compared with blood donors (Fig 7C: P<0.0001). The TK1 activity levels were also significantly higher in T2 (n = 31; mean ± SD = 2.30±1.40 and median = 2.1 pmol/min/mL), and T3 (n = 11; mean ± SD = 6.40±9.80 and median = 2.6 pmol/min/mL) compared with blood donors (Fig 7D: P<0.0001). Furthermore, the sera from metastasized breast cancer patients had significantly higher levels of TK1 protein and TK1 activity compared to patients without metastasis (Fig 7E and 7F).

#### Patients with prostate cancer

**Fig 8:**
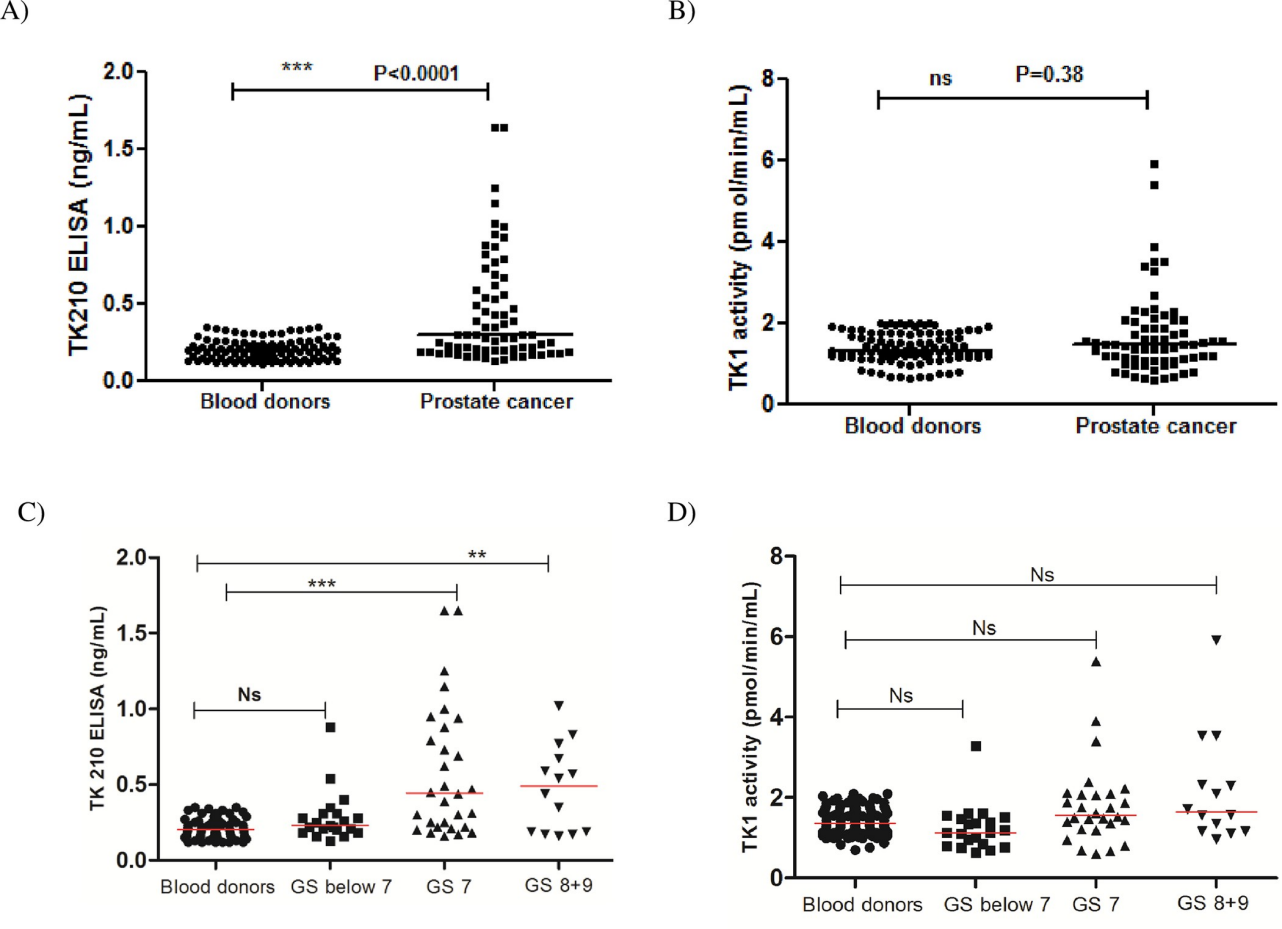
TK1 protein and TK1 activity values in sera from patients with prostate cancer. (A) TK1 protein values in sera from blood donors (●) and patients with prostate cancer (■). (B) TK1 activity levels in sera from blood donors (●) and patients with prostate cancer (■). (C) Comparison of TK1 protein levels in sera from blood donors (●), GS below 7 (■), GS 7(▲) and GS 8+9 (▼) prostate cancer patients. (D) Comparison of TK1 activity levels in sera from blood donors (●), GS below 7 (■), GS 7(▲) and GS 8+9 (▼) prostate cancer patients. The error bars denote median.

We tested 70 serum samples from patients with prostate cancer. The TK1 protein levels in prostate cancer sera ranged from 0.14 to 1.64 ng/mL (mean ± SD = 0.44±0.33 and median = 0.30) and were significantly higher compared to blood donors (Fig 8A). Even though some sera had high TK1 activity levels but there was no significant difference in mean TK1 activity values in sera from prostate cancer patients (mean ± SD = 1.69±1.00 pmol/min/mL and median = 1.47) compared with levels in sera from blood donors (Fig 8B). There was no significant correlation between TK1 protein (or) TK1 activity in relation to age (S3 File). Based on the Gleason score (GS), prostate serum samples were divided into 3 categories: GS below 7 (well differentiated), GS 7 (moderately differentiated) and GS 8 + 9 (poorly differentiated). The TK1 protein levels in GS 7 (n = 30; mean ± SD = 0.58±0.43 and median = 0.45 ng/mL) and GS 8 + 9 (n = 11; mean ± SD = 0.48±0.28 and median = 0.49 ng/mL) were significantly higher compared to blood donors (Fig 8C, P<0.0001). In contrast, there was no significant differences in serum TK1 activity between GS below 7, GS 7 and GS 8 + 9 compared to blood donors (Fig 8D).

#### Comparison between assays

**Fig 9:**
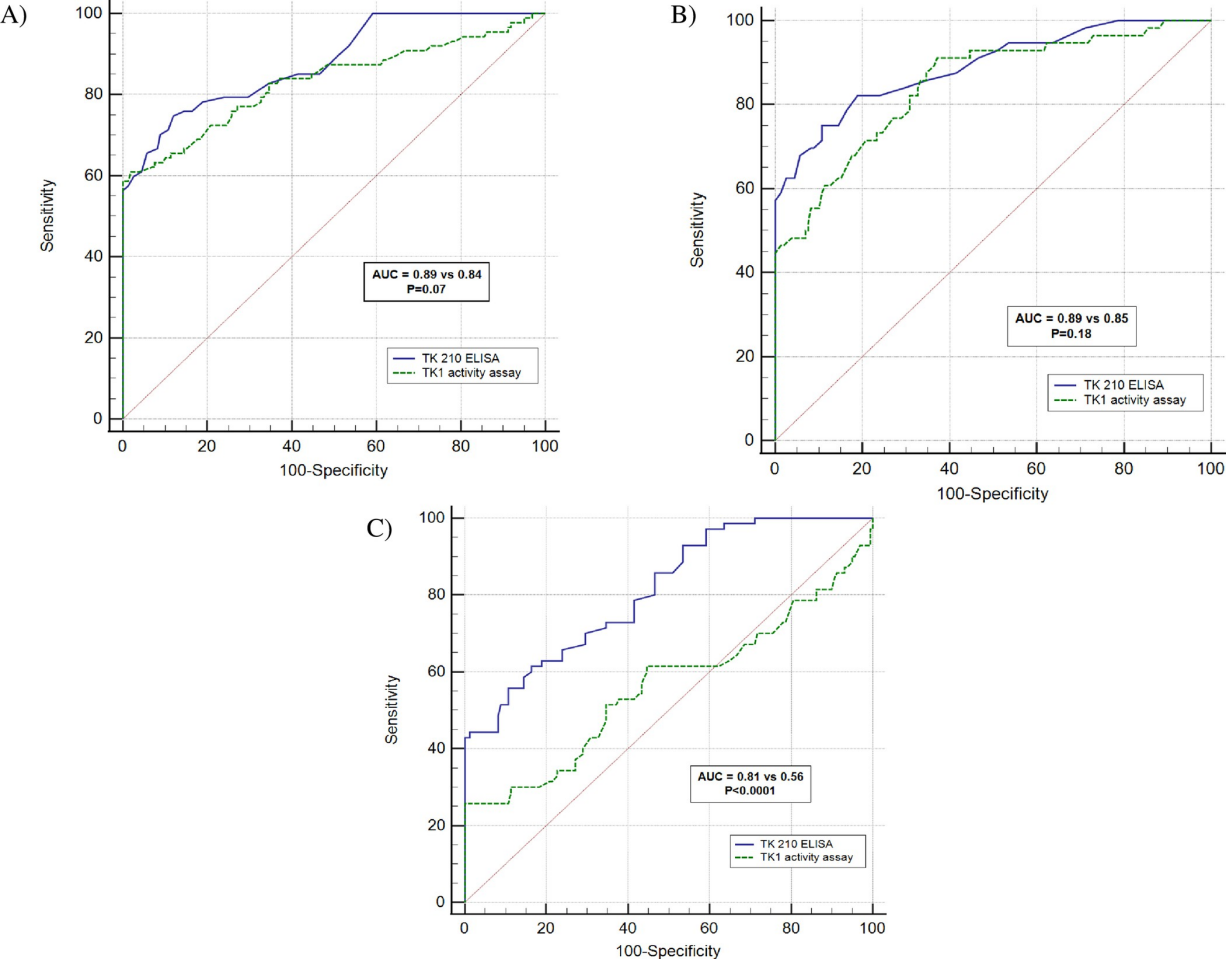
Receiver operating characteristic (ROC) curve comparison between two assays in different malignancies. (A) Comparison of ROC curves for TK 210 ELISA and TK1 activity assay in differentiation of hematological malignancies from blood donors. (B) Comparison of ROC curves for TK 210 ELISA and TK1 activity assay in differentiation of breast cancer from blood donors. (C) Comparison ROC curves analysis of TK 210 ELISA and TK1 activity assay in sera from blood donors and prostate cancer patients.

The performances of assays were compared by using ROC curve analysis with data from different malignancies (Table 5). In hematological malignancies comparative ROC curve analysis showed that the TK 210 ELISA (sensitivity = 62%) had similar sensitivity to the TK1 activity assay (sensitivity = 59%) at a specificity of 98% (Fig 9A). Similar analysis on breast cancer sera showed that the TK 210 ELISA had a higher sensitivity (sensitivity = 59%) compared to the TK1 activity assay (sensitivity = 47%) at a specificity of 98% although the difference was not statistically significant between the area under curves (Fig 9B). However, the TK 210 ELISA (sensitivity = 44%) showed significantly higher sensitivity compared to the TK1 activity assay (sensitivity = 25%) in differentiation of prostate cancer sera compared to of blood donors (Fig 9C) at a specificity of 98%.

**Table 5:** ROC curve analysis of TK 210 ELISA and TK1 activity assay for different malignancies at a specificity of 98%.

Parameter	No of sera	cut-off	No of sera above cut-off	AUC	Sensitivity	95% CI	+LR (95% CI)	-LR (95% CI)
**Hematological tumors**	100							
TK 210 ELISA		0.34 ng/mL	62	0.89	62	51.7–71.5	49.3 (12.3–197)	0.38 (0.3–0.5)
TK1 activity assay		2 pmol/min/mL	59	0.84	59	47.6–69.1	46.6 (11.6–187)	0.42 (0.3–0.5)
**Breast cancer**	56							
TK 210 ELISA		0.34 ng/mL	33	0.89	59	45.0–71.9	46.8 (11.6–188)	0.42 (0.3–0.6)
TK1 activity assay		2 pmol/min/mL	27	0.85	47	33.0–60.3	36.9 (9–150)	0.54 (0.4–0.7)
**Prostate cancer**	70							
TK 210 ELISA		0.34 ng/mL	32	0.81	44	31.1–55.3	34.1 (8–139)	0.58 (0.5–0.7)
TK1 activity assay		2 pmol/min/mL	19	0.56	25	16.0–35.6	2.41 (1.3–4.4)	0.83 (0.7–1.0)

AUC: area under curve, +LR = positive likely hood ratio and–LR = negative likely hood ratio.

**Fig 10:**
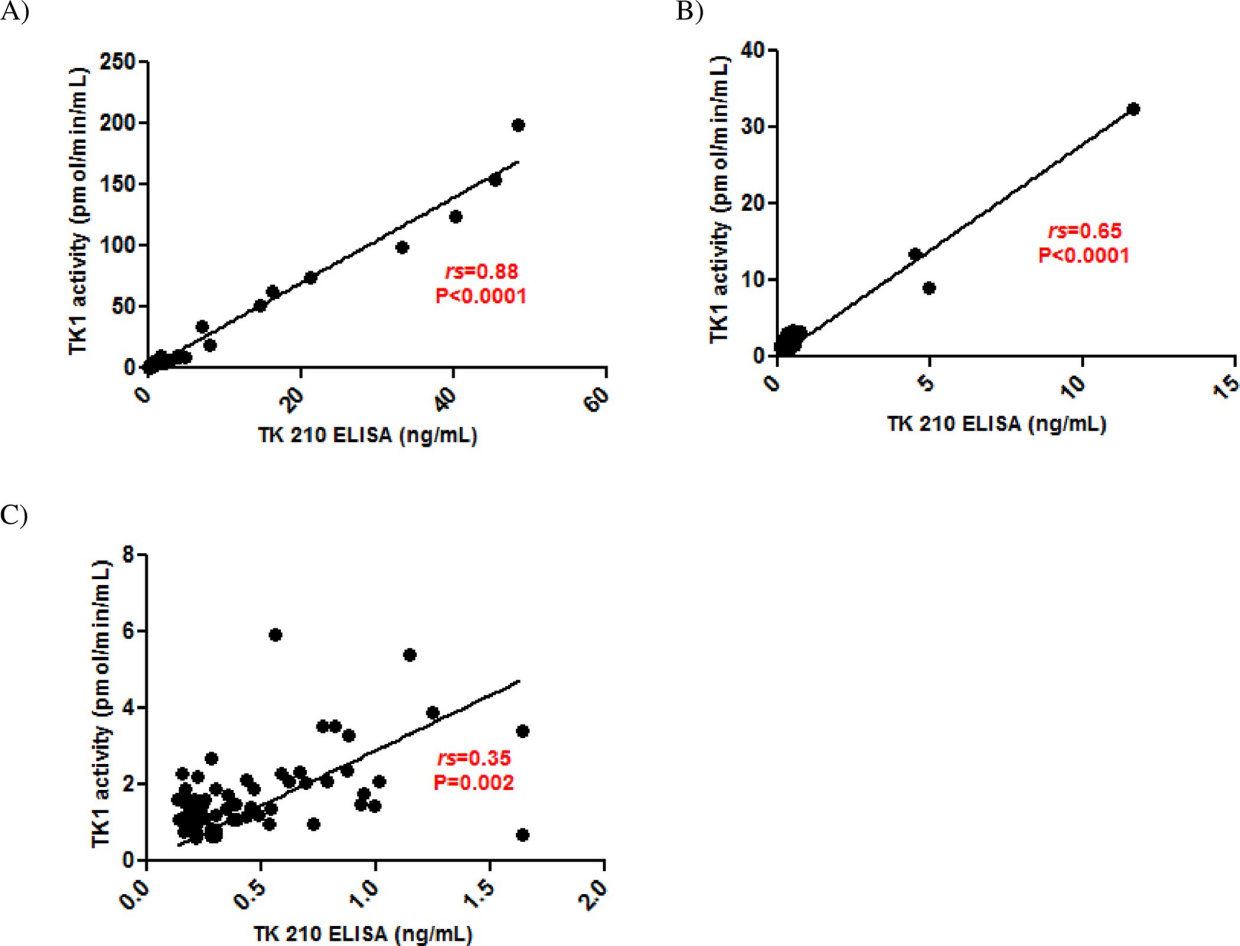
Correlation between TK 210 ELISA and TK1 activity assays for different malignancies. (A) hematological malignancies (B) breast cancer (C) prostate cancer.

A Passing-Bablok regression analysis of the TK 210 ELISA (y) and TK1 activity assays for all patient groups gave a correlation coefficient of 0.72 (P<0.0001; 95 CI-0.65 to 0.77) with an equation of y = 0.457+ 3.50 x (n = 226). However, the correlations varied depending on the type of malignancy; hematological malignancies, breast, and prostate cancer, respectively (*r*_*s*_ = 0.88, *P<*0.0001; Fig 10A, *rs* = 0.65, *P*<0.0001; Fig 10B and *rs* = 0.35, *P =* 0.002; Fig 10C).

## Discussion

Here, we describe the development and characterization of a sandwich ELISA for TK1 protein determinations using sera from blood donors and patients with malignant diseases. The precision, reproducibility and recovery of the AroCell TK 210 ELISA were sufficient for measurement of TK1 protein concentrations in sera from most individuals with a detection limit of 0.12 ng/mL. Attempts have been made earlier to develop an ELISA for serum TK1 protein determination using monoclonal or polyclonal antibodies [23, 24] but to our knowledge there is no fully validated and CE marked assay for this purpose available. Highly complex forms of TK1 have been observed in sera from healthy and subjects with viral infections and malignant diseases [13, 19 and 21]. Determining the conditions needed to reduce the complexity of serum TK1 and specifically designed and selected monoclonal anti-TK1 antibodies were key factors for the development of the AroCell TK 210 ELISA. Moreover, the reducing and stabilizing components in SDB limits unspecific binding and increase the exposure of the epitopes for the antibodies as demonstrated in previous studies [13, 19, 21]. Furthermore, a recent study on the production and characterization of TK1 antibodies against several different TK1 epitopes also showed that the antibodies against the C-terminal region of TK1 results in ELISAs with higher sensitivity for serum TK1 detection than antibodies against other regions [25] in accordance with the results presented here.

Experiments showing linearity with high serum TK1 concentrations were performed to demonstrate proportionality between serum TK1 and the recombinant TK1 used as calibrators. Inorder to obtain a linear relationship betweenthe TK1 calibrator and the serum TK1 concentrations in the TK 210 ELISA a serum matrix was required. Intra- and inter assay variations were determined with the calibrators and with serum samples and similar ranges of variations were observed. These parameters in terms of accuracy and imprecision meet the acceptance criteria for ELISA according to CLSI guidelines. When sample stability was tested, there was no alterations in the TK1 protein concentrations after four freeze-thaw cycles and samples stored at -80°C were stable for at least 1 year.

The assay has been validated with human serum samples but further validation studies are necessary for the measurements of TK1 concentrations in case of lithium heparin plasma, although preliminary results strongly indicate that these type of patient samples can be used with the AroCell TK 210 ELISA. Other preliminary experiments demonstrate that EDTA plasma give aberrant results with the TK 210 ELISA. In blood donors, no significant sex difference was observed in the age group between 22 to 80 years. However, in a recent study where TK1 protein levels in a large group of apparent healthy donors (n = 264) were determined, a significant difference was found between men and women. However, the upper limit of TK1 protein levels in blood donors (95% CI) in that study was found to be 0.40 ng/mL, which is not significantly different than that reported here (0.34 ng/mL) [26]. In addition, the study also showed that results obtained with the AroCell TK 210 ELISA had significant correlation with other activity based assays [26]. Here a similar comparison was made between a subset of serum samples with AroCell TK 210 ELISA and TK1 activity based assay. Overall, a significant correlation between the assays (*rs* = 0.72) was found, the correlation in the case of hematological malignancies was (*rs* = 0.88) higher compared to what was observed with sera from pateints with breast and prostate cancer (*rs* = 0.65 and *rs* = 0.35, respectively). Another recent study, where comparison of TK 210 ELISA and TK-Liaison was done in different types of malignancies. The assay performance differed depending on the type of malignancy, in case of hematological malignancies both the assays had similar sensitivity whereas in case of solid tumors, TK 210 ELISA showed higher sensitivity compared to TK-Liaison. In addition, a significantly high correlation was found between TK 210 ELISA and TK-Liaison for hematological malignancies (rs = 0.95) compared to solid tumors (rs = 0.50) [27]. The lower correlation of the AroCell TK 210 ELISA and TK1 activity assays in the latter samples is most likely due to the fact that TK 210 ELISA measures both active as well as inactive forms of TK1 in sera from solid tumor patients whereas the TK1 activity assay measures only the active TK1. The analytical sensitivity of the TK 210 ELISA was sufficient to measure TK1 protein in more than 85% of the blood donors but approx 15% were below the LOD.

The present study demonstrated significant increase in serum TK1 protein levels in subjects with different malignancies compared to in blood donors,but there was an overlap in the distribution of the TK1 concentrations between the different populations. There are published studies describing the performance of the AroCell TK 210 ELISA in breast cancer and prostate cancer patients. These studies also showed that the TK 210 ELISA could complement tumor specific biomarkers such as CA 15–3 and *pro*PSA in the management of breast and prostate cancer patients [28, 29]. Another recent study showed that AroCell TK 210 ELISA is useful in *in-vitro* studies to measure the changes in cellular and extracellular TK1 concentrations in cell toxicity studies, particularly with the drugs targeting cell proliferation and DNA damage [30]. An advantage of the AroCell TK 210 ELISA is that it does not cross react with the TK2 protein leading to greater specificity for TK1 protein determinations in clinical samples. Furthermore, the AroCell TK 210 ELISA does not cross-react with mouse TK1 enabling the TK 210 ELISA to be used in drug development projects involving mouse xenograft models. These studies demonstrate the potential use of the AroCell TK 210 ELISA as a tool in drug development.

The AroCell TK 210 ELISA has several advantages such as shorter run time of the assay (compared to some activity-based assays), no need for special training and equipment, and there is no radioactive material involved and the assay is easily compatible to automated platforms.

### Conclusions

The validation studies described here on the AroCell TK210 ELISA demonstrate that it is a suitable method for the quantification of the TK1 protein in the human serum and lithium heparin-plasma. The AroCell TK 210 ELISA sandwich format provides high specificity and sensitivity for TK1 protein determinations with a broad dynamic range, which give this biomarker the capacity to be a valuable tool in the clinical management of patients with different malignancies and in drug development.

## Supporting information

S1 File Interfering substances evaluation with TK 210 ELISA. (PDF)

S2 File TK1 activity and TK1 protein distribution in men and women with haematological malignancies. (TIF)

S3 File Age based comparison of TK1 activity, TK1 protein in breast cancer patients and prostate cancer patients. (TIF)
