# Supplementary material for: Analytical and clinical characterization of an optimized dual monoclonal sandwich ELISA for the quantification of thymidine kinase 1 (TK1) protein in human blood samples
Source: PLoS One. 2022 Oct 6;17(10):e0275444. doi: 10.1371/journal.pone.0275444 (PMC9536554; doi:10.1371/journal.pone.0275444)
Supplement: S1 File — (PDF) [file pone.0275444.s001.pdf]

Interfering substances

Four TK1 positive serum pools (S1, S2 are naïve TK1 sera. S3, S4 are recombinant TK1) were spiked with 20 mg/dL of bilirubin, 300 mg/dL of Glyceryl triolate, 400/ 200 mg/dL of hemoglobin and 60 mg/mL of HSA. Addition of hemoglobin and HSA demonstrated significant deviation (>10%) in the percentage of recovery (shown in red color in supplementary table 1). This result indicated potential interference by hemoglobin and high HSA in TK1 protein determination using TK 210 ELISA. However, 60 mg/ml is above the upper limit of normal serum levels HSA. The interference by hemoglobin suggests that hemolysed samples are not recommended for TK1 analysis by TK 210 ELISA.

Supplementary Table 1:

Interference substances	% of recovery(+) vs (-)
20 mg/dL bilirubin	S1 - 97%<br>S2 - 97%<br>S3 - 101%<br>S4 - 100%
300 mg/dL Glyceryl triolate	S1 - 102%<br>S2 - 99%<br>S3 - 95%<br>S4 - 98%
400 mg/dL hemoglobin	S1 - 113%<br>S2 - 114%<br>S3 - 100%<br>S4 - 105%
200 mg/dL hemoglobin	S1 - 113%<br>S2 - 105%<br>S3 - 106%<br>S4 - 102%
60 mg/mL HSA	S1 - 101%<br>S2 - 116%<br>S3 - 80%<br>S4 - 85%
