# Supplementary figures and images for: Analytical and clinical characterization of an optimized dual monoclonal sandwich ELISA for the quantification of thymidine kinase 1 (TK1) protein in human blood samples
Source: PLoS One. 2022 Oct 6;17(10):e0275444. doi: 10.1371/journal.pone.0275444 (PMC9536554; doi:10.1371/journal.pone.0275444)

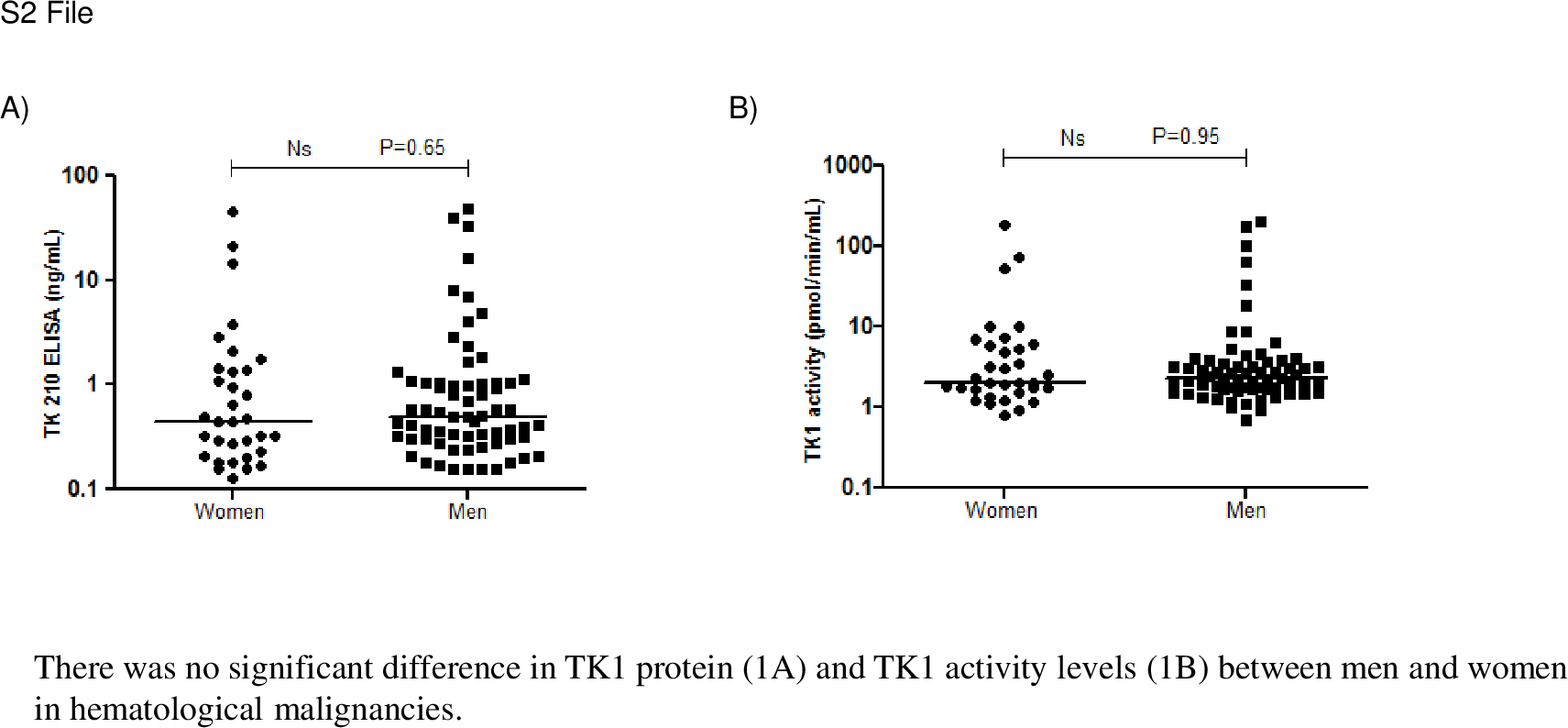

Supplement: S2 File — (TIF) [file pone.0275444.s002.tif]

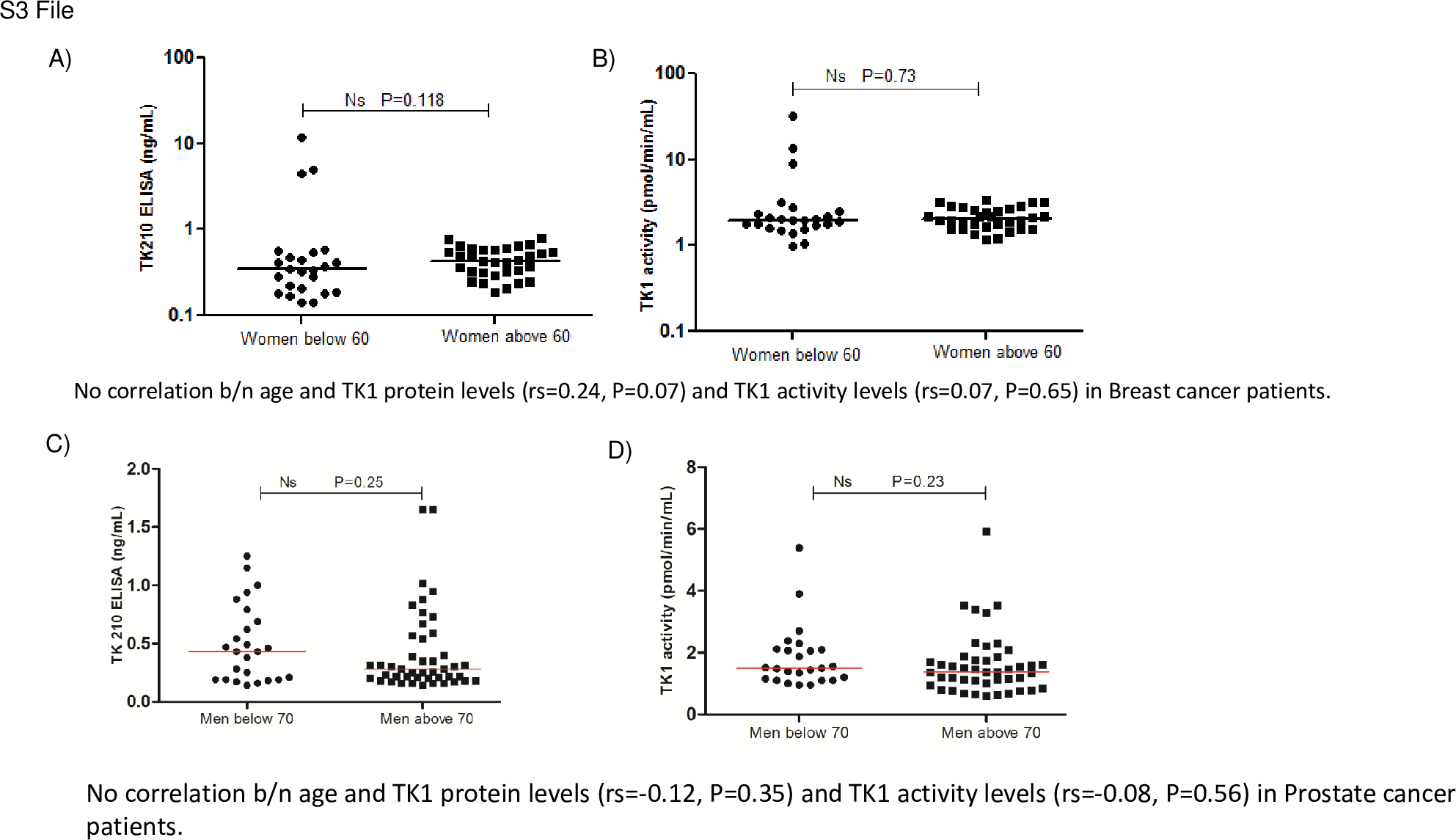

Supplement: S3 File — (TIF) [file pone.0275444.s003.tif]
